# Adapting Learning Activity Selection to Emotional Stability and Competence

**DOI:** 10.3389/frai.2020.00011

**Published:** 2020-03-24

**Authors:** Manal Alhathli, Judith Masthoff, Nigel Beacham

**Affiliations:** ^1^Department of Computing Science, University of Aberdeen, Aberdeen, United Kingdom; ^2^Vice-Rectorate of Development and Quality, Princess Nourah Bint Abdul Rahman University, Riyadh, Saudi Arabia; ^3^Department of Information and Computing Sciences, Utrecht University, Utrecht, Netherlands

**Keywords:** learning, adaptation, educational recommender, competency, emotional stability, personalization

## Abstract

This paper investigates how humans adapt next learning activity selection (in particular the knowledge it assumes and the knowledge it teaches) to learner personality and competence to inspire an adaptive learning activity selection algorithm. First, the paper describes the investigation to produce validated materials for the main study, namely the creation and validation of learner competence statements. Next, through an empirical study, we investigate the impact on learning activity selection of learners' emotional stability and competence. Participants considered a fictional learner with a certain competence, emotional stability, recent and prior learning activities engaged in, and selected the next learning activity in terms of the knowledge it used and the knowledge it taught. Three algorithms were created to adapt the selection of learning activities' knowledge complexity to learners' personality and competence. Finally, we evaluated the algorithms through a study with teachers, resulting in an algorithm that selects learning activities with varying assumed and taught knowledge adapted to learner characteristics.

## 1. Introduction

Intelligent Tutoring Systems extend the traditional information-delivery learning system by considering learners' characteristics to improve the effectiveness of a learner's experience (Brusilovsky, 2003). Whilst traditional e-learning has contributed to flexibility in learning and reduced education costs, ITS attempt to fit the particular needs of each individual (Park and Lee, 2004; Brusilovsky and Millán, 2007; Siddappa and Manjunath, 2008; Dascalu et al., 2016).

Adapting ITS to individual learner characteristics helps learners to achieve learning goals and supports personalized learning (Brusilovsky, 1998a,b; Ford and Chen, 2000; Drachsler et al., 2008a; Santos and Boticario, 2010). Several studies have shown that the main problem with traditional e-learning is the lack of learner satisfaction due to delivering the same learning experience to all learners, irrespective of their prior knowledge, experience, and preferences (Ayersman and von Minden, 1995; Cristea, 2003; Rumetshofer and Wöß, 2003; Stewart et al., 2005; Di Iorio et al., 2006; Sawyer et al., 2008). Researchers have tried to address this dissatisfaction by attempting to personalize the learning experience for the learner. A personalized learning experience can help to improve learner satisfaction with the learning experience, learning efficiency, and educational effectiveness (Brusilovsky, 2001; De Bra et al., 2004; Huang et al., 2009). Most research on adaptive learning interaction shows an increase in learning outcomes (Anderson et al., 1995; Vandewaetere et al., 2011).

An important aspect of adaptive e-learning is the adaptive selection of learning activities. In fact, the main goal of adaptive e-learning was identified by Dagger et al. (2005) as “*e-learning content, activities and collaboration, adapted to the specific needs and influenced by specific preferences of the learner and built on sound pedagogic strategies*.” Studies have confirmed that the role of adaptation in e-learning is to improve the instruction content given to heterogeneous learner groups (Brusilovsky et al., 1998; Seters et al., 2011). Personalizing the selection of learning activities is needed to make learning more efficient (Camp et al., 2001; Salden et al., 2004; Kalyuga and Sweller, 2005).

Previous studies show that adaptive activity selection impacts factors, such as attitude and behavior (Ones et al., 2007), skills acquisition (Oakes et al., 2001), and productivity (Judge et al., 1999; Bozionelos, 2004). Considering individual differences among learners will improve learning achievement (Shute and Towle, 2003; Tseng et al., 2008). Personalized activity selection yields more effective and efficient learning outcomes, with researchers reporting a positive effect on learners' motivation and learning efficiency (Schnackenberg and Sullivan, 2000; Corbalan et al., 2008).

Learning is influenced by both characteristics of the learner (such as expertise, abilities, attitudes, performance, mental effort, personality) and characteristics of the learning activity (LA) (such as LA complexity, LA type, amount of learner support provided) (Lawless and Brown, 1997; Zimmerman, 2002; Salden et al., 2006; Okpo et al., 2018).

Previously, in six focus group studies (Alhathli et al., 2018b), we investigated what type of LAs to select for a particular learner. Results showed a clear impact of personality (self-esteem, openness to experience, emotional stability) on the use of prior knowledge and topics taught in LA selection. Focus group participants mentioned several other factors that should be considered when selecting a LA, such as a learner's academic record and ability and the LA's difficulty. Given the focus group results, we decided to investigate the impact of Emotional Stability (ES) and learners' competence on the selection of the next LA. In particular, this paper investigates the impact on the selected LA content: both the knowledge taught by the LA and the prior knowledge it uses. The LA style (e.g., visual vs. textual) is not included in this study, as we studied the impact of personality on the selected LA style before (Alhathli et al., 2018a).

## 2. Background and Related Work

### 2.1. Learner Characteristics to Adapt to

Researchers have shown an increased interest in adapting to learner characteristics, such as personality traits, motivation, performance, cognitive efficiency, needs, and learning style (Miller, 1991; Wolf, 2003; Shute and Zapata-Rivera, 2007; Schiaffino et al., 2008; Komarraju et al., 2011; Vandewaetere et al., 2011; Richardson et al., 2012; Alhathli et al., 2016, 2017; Dennis et al., 2016; Okpo et al., 2016, 2017). There is considerable debate around which learner characteristics are worth modeling more than others^1^. Vandewaetere et al. (2011) classified individual characteristics into three groups:

- Cognitive, which is a collection of cognition related characteristics, such as the previous knowledge of the learner (Graesser et al., 2007), learners' abilities (Lee and Park, 2008), learning style (Germanakos et al., 2008), and learning objectives (Kelly and Tangney, 2002);
- Affective, which is a collection of feeling related attributes, such as learner mood (Beal and Lee, 2005b), self-efficacy (Mcquiggan et al., 2008), disappointment (Forbes-Riley et al., 2008) and confusion (Graesser et al., 2008); and
- Behavior, whereby a learner behaves differently when they are interacting with computers. These behavioral characteristics can be related to the need for help or feedback (Koutsojannis et al., 2001), the degree of self-regulated learning (Azevedo, 2005), and the number of attempts, tasks and learner experience (Hospers et al., 2003).

In our classification in Table 1, we broadened the affective category to psychological aspects, including also personality traits, motivation, and mental effort. We extended the cognitive category to include cognitive style as distinct from learning style. We renamed the behavioral category to include performance. We added an additional category called personal information for learner characteristics not covered by the classification of Vandewaetere et al. (2011), such as demographics and cultural background. Table 1 shows examples of research into adapting to these learner characteristics.

**Table 1:** Examples of learner characteristics adapted to in adaptive educational systems.

**Learner characteristics adapted to**		**Example research**
	Personality; Big 5	Robison et al., 2010; Nunes and Hu, 2012; Alhathli et al., 2016, 2018b; Dennis et al., 2016
Psychological aspects	Self-efficacy	Beal and Lee, 2005a; Mcquiggan et al., 2008
	Mental effort	Salden et al., 2006; Okpo et al., 2018
	Motivation	Beal and Lee, 2005a; Cocea and Weibelzahl, 2006
	Affective state and mood	Beal and Lee, 2005b; Forbes-Riley et al., 2008; Graesser et al., 2008; Odo et al., 2018
Behavior and performance	Learner competence	Mitrović et al., 1996; Davidovic et al., 2003; Tsiriga and Virvou, 2004; Cheng et al., 2008; Corbalan et al., 2008
	Problem solving skills	Melis et al., 2001; Pholo and Ngwira, 2013
	Help seeking behavior and self-regulated learning	Koutsojannis et al., 2001; Azevedo, 2005
	Learner progress	Brusilovsky et al., 1996; Revilla et al., 2008; Trotman and Handley, 2008; Verdú et al., 2012
Cognition	Cognitive style	Triantafillou et al., 2004; Graesser et al., 2007; Mampadi et al., 2011; Lo et al., 2012; Alhathli et al., 2018a
	Knowledge state and prior knowledge	Shute, 1995; Ray and Belden, 2007; Kelly, 2008; Petrovica, 2013
	Learning style	Magoulas et al., 2003; Sun and Cheng, 2007; Germanakos et al., 2008; Latham et al., 2012; El-Bishouty et al., 2014; Alhathli et al., 2017
	Learner objectives	Kelly and Tangney, 2002; Vassileva and Bontchev, 2006
Personal information	Learner profile, demographics, cultural background, and preferences	Hwang, 1998; Widyantoro et al., 1999; Chang et al., 2000; Sugiyama et al., 2004; Reategui et al., 2008; Adamu Sidi-Ali et al., 2019

The learner characteristics investigated in this paper are Personality and Prior knowledge and Competence.

#### 2.1.1. Personality

Previous studies have acknowledged that both personality and general cognitive ability influence learners' performance (Ree et al., 1994; Barrick et al., 2001; Barrick, 2005). It has been suggested that there is a convincing relation between personality and other factors, such as attitude and behavior (Ones et al., 2007), skill acquisition (Oakes et al., 2001), and productivity (Judge et al., 1999; Bozionelos, 2004). Personality can be defined as the individual differences in people's emotional, interpersonal, experiential, attitudinal and motivational styles (John and Srivastava, 1999). Researchers have shown an increased interest in adapting to personality traits (Miller, 1991; Komarraju et al., 2011; Richardson et al., 2012; Dennis et al., 2016; Okpo et al., 2016, 2017).

The most adopted model of personality is the Five-Factor Model (also known as the Big Five), which is based on five dimensions (Costa and McCrae, 1992a, 1995): (i) extroversion, (ii) agreeableness, (iii) conscientiousness, (iv) emotional stability, (v) openness to experience (McCrae, 1992). Extroversion refers to a higher degree of sociability, energy, assertiveness, and talkativeness. Emotional stability refers to the opposite of neurotism, i.e., someone who is calm and not easily upset. Openness to experience refers to those who are interdependent-minded, and intellectually strong. Conscientiousness refers to being disciplined, organized, and achievement-oriented. Finally, Agreeableness refers to being good-natured, helpful, trustful, and cooperative (Miller, 1991). These traits have been found across all cultures (McCrae and Costa, 1997; Salgado, 1997). In addition, these traits are relatively stable over time (Costa and McCrae, 1992b).

Several studies have shown the effect of personality on the learning process, and it has been investigated that certain personality traits consistently correlate with learner achievement, motivation, and success (Komarraju and Karau, 2005; Poropat, 2009; Clark and Schroth, 2010; Komarraju et al., 2011; Hazrati-Viari et al., 2012; Richardson et al., 2012).

#### 2.1.2. Prior Knowledge and Competence

Numerous terms have been used to refer to prior knowledge (e.g., current knowledge, expert knowledge, personal knowledge, and experiential knowledge) (Dochy, 1992, 1994). Interest in a learner's prior knowledge has appeared in many educational studies. An individual's prior knowledge is considered as a set of skills, or abilities that are present in the learning process (Jonassen and Grabowski, 1993; Shane, 2000). Previous investigations have demonstrated the potential impact of prior knowledge on cognitive processes, with positive and significant effects on learner's performance, abilities, and achievement (Byrnes and Guthrie, 1992; Dochy, 1994; Gaultney, 1995; Thompson and Zamboanga, 2003).

In our focus groups, we found that prior knowledge impacts the selection of the next LA. Thus, we decided to use learners' competence in terms of learner knowledge and ability.

Competence can be defined differently depending on the discipline. The dictionary defines competence as a condition or as quality of effectiveness. Competence refers to an individual's capability, sufficiency, ability, and successes. A large amount of competence research refers to the skills and requirements needed for a particular task or profession (Willis and Dubin, 1990; Parry, 1996). Competence is seen as a reflection of multiple concepts, such as performance. Competence and performance are related, with competence depicting the mean of better performance (Klemp, 1979; Woodruffe, 1993).

However, performance can be affected by other factors, such as motivation and effort (Schambach, 1994). Competencies are also considered as a core component of goal achievement. Achievement goals are defined as a cognitive representation of a competence efficiency and ability that an individual seeks to obtain (Elliot, 1999; Bong, 2001; Elliot and McGregor, 2001). Competence can be defined depending on the standard or referent that is used in evaluation (Elliot and Thrash, 2001). Competence may be evaluated according to three different standards, as follows: (1) absolute, the requirement of the task itself; (2) intra-personal, past or maximum attainment; and (3) normative, the performance of others (Butler, 1988; Elliot and McGregor, 2001).

### 2.2. Educational System Characteristics to Adapt

Many aspects of an educational system can be adapted to a learner. For example, Masthoff (1997) argued for adapting navigation through the course content, exercise selection, feedback, instructions, provision of hints, and content presentation. For example, feedback has been adapted to performance and personality (Dennis et al., 2016) and culture (Adamu Sidi-Ali et al., 2019), difficulty level to performance, personality and effort (Okpo et al., 2018), navigational control to learner goals and knowledge (Masthoff, 1997), and learning content and presentation to learning styles (Bunderson and Martinez, 2000).

This paper focuses on adaptive learning activity selection. Table 2 provides examples of adaptive educational systems that include adaptive LA selection, the learner characteristics used to guide the adaptation, and the system control used to provide the adaptation. The following types of system control can be distinguished:

1. *Curriculum Sequencing* provides learners with a planned sequence of learning contents and tasks (Brusilovsky, 2003),
2. *Adaptive Navigation Support* helps learners to find their paths in the learning contents according to the goals, knowledge, and other characteristics of an individual learner (Brusilovsky, 1996),
3. *Collaborative Filtering* (and other educational recommender systems' techniques) supports learners to find learning resources that are relevant to their needs and interests (Recker and Walker, 2003; Recker et al., 2003; Schafer et al., 2007; Drachsler et al., 2008a),
4. *Adaptive Presentation* supports learners by providing individualized content depending on their preferences, learning style and other information stored in the learner model (Beaumont and Brusilovsky, 1995).

**Table 2:** Examples of adaptive educational systems that adapt content selection.

**System**	**Learner characteristics**	**System control**
CDG (Vassileva, 1997)	Personal traits; learning goal; preferences	Curriculum sequencing
AST (Specht et al., 1997)	Knowledge level; learning style preferences	Curriculum sequencing
KBS hyperbook (Henze et al., 1999)	Knowledge level; learning goals	Adaptive navigation support
Arthur (Gilbert and Han, 1999)	Learning style preferences	Curriculum sequencing
Altered Vista (Recker and Walker, 2003)	Preferences	Collaborative filtering
RACOFI (Anderson et al., 2003)	Multidimensional ratings	Collaborative filtering
INSPIRE (Papanikolaou et al., 2001)	Knowledge level; learning style	Adaptive presentation
Learning object sequencing (Shen and Shen, 2004)	Knowledge base; learner competence	Curriculum sequencing
QSIA (Rafaeli et al., 2004)	Knowledge sharing	Collaborative filtering
Rmashed (Drachsler et al., 2008b)	Learning goals	Collaborative filtering
CYCLADES (Avancini and Straccia, 2005)	User interests; preferences	Collaborative Filtering
Rmashed (Drachsler et al., 2008b)	Learning goals	Collaborative filtering
iLearning (Wang et al., 2014)	Knowledge level	Collaborative filtering

The LA selection in this paper is concerned with selecting activities that are well-suited to learners' personality, prior knowledge and competence. This is related to Adaptive Navigation and Educational Recommender Systems, given a LA is selected as most suitable for a learner based on the knowledge the LA assumes and teaches. The LA selected by the system can be used to support learners in finding what LA to do next or by an ITS to automatically present that LA.

The domain model in our studies contains the LAs, and in particular their topics and the type and quantity of knowledge they use and produce.

#### 2.2.1. Learning Activity Topic

Educational systems which recommend or provide personalized learning contents often require information about the topics covered in the learning materials, courses, and assignments it selects from (Liang et al., 2006; Soonthornphisaj et al., 2006; Prins et al., 2007; Yang and Wu, 2009; Ricci et al., 2011). Bloom's Taxonomy defines three overarching domains of LAs: Cognition (e.g., teaching mental skills), Affective (e.g., teaching attitudes), and Psychomotor (teaching manual of physical skills) (Bloom, 1956). This paper focuses on the cognitive domain. Within the cognitive domain, there are many sub-domains. For example, educational recommender systems have been developed for programming (Mitrovic et al., 2002; Wünsche et al., 2018) and learning languages (Hsu, 2008; Wang and Yang, 2012). Even within such a sub-domain, multiple topics exist. For example, when teaching somebody English, one could have a LA on ordering food, and a different activity on buying groceries. Educational recommender systems often select based on learner interests, so need detailed information on the topics covered in a LA.

#### 2.2.2. Learning Activity Knowledge

Incorporating learner characteristics, such as the learner's knowledge, interests and goals in an adaptive educational system is a well-established approach discussed by Brusilovsky (2003, 2007). To adapt LA selection, a match needs to be made between the learner's knowledge, goals and interests and what LAs have to offer and require. In traditional education, LAs are often described in terms of prerequisites (the knowledge required of a learner to participate in a LA) and learning outcomes (the knowledge the learner will gain by successfully completing a LA) (Anderson et al., 2001).

## 3. Creation and Validation of Learner Competence Statements

This section describes the development and validation of competence statements used in later studies. Many statements can be used to describe different levels of competency, but no existing list clearly defined varying levels of individual competence. Initially, 26 statements were produced to cover five categories of learners' competence. All statements are commonly used to depict different competence levels. Table 3 shows the resulting statements and their initial categorization. These statements will be used in our investigations on the impact of personality and competence on the selection of LA.

**Table 3:** Competence statements (grouped by initial categorization) mapped to competence rating.

**Initial**	**Competence rating by participants %**												**Average**	
**Cat**.	**Statement**	**1**	**2**	**3**	**4**	**5**	**6**	**7**	**8**	**9**	**10**	**Rating**	**Median**
A	No	95%	5%									1.06	1.00
	**Very low**	28%	72%									1.71	2.00
	Poor	17%	50%	28%	5%							2.22	2.00
	Hardly any	28%	50%	11%			11%					2.28	2.00
B	Little		22%	50%	28%							3.06	3.00
	Low	5%	11%	62%	17%	5%						3.06	3.00
	Limited	5%	17%	28%	34%		11%			5%		3.72	3.50
	Slight		5%	11%	39%	28%	17%					4.39	4.00
	Some			23%	39%	5%	28%		5%			4.61	4.00
C	Fair	5%		5%	5%	28%	39%	18%				5.33	6.00
	Quite some				28%	17%	22%	22%	11%			5.72	6.00
	Medium					56%	22%	17%	5%			5.72	5.00
	**Moderate**				11%	39%	17%	28%		5%		5.83	5.50
	Standard					39%	17%	22%	22%			6.28	6.00
D	Good				5%	28%		34%	33%			6.61	7.00
	Sufficient					5%	28%	56%	11%			6.72	7.00
	Recognized				5%	17%	17%	34%	5%	5%	17%	6.76	7.00
	Much				5%	5%	5%	18%	28%	39%		7.00	8.00
	Very good				5%		16%	11%	28%	40%		7.72	8.00
	High							28%	50%	17%	5%	8.00	8.00
	Advanced						5%	23%	33%	28%	11%	8.17	8.00
E	Very high						5%	17%	5%	34%	39%	8.83	9.00
	Excellent								22%	22%	56%	9.23	10.00
	Full								16%	28%	56%	9.39	10.00
	**Outstanding**								5%	39%	56%	9.50	10.00
	Extreme						5%				95%	9.78	10.00

*Statements in bold were used in the follow-on study*.

### 3.1. Study Design

#### 3.1.1. Participants

Thirty participants (staff and students of the university) completed an on-line survey (7% aged 18–25, 53% 26–35, 40% 36–45), which took about 15 min to complete. The data from 18 participants were used for the final analysis (9 male, 9 female). The others were excluded due to the low quality of their responses: either straight-lining (giving the same answer to all statements), or not putting “No competence” toward the bottom of the scale as directed in the explanation.

#### 3.1.2. Statement Validation

Participants were shown 26 statements, and rated how much they felt these statements reflect the individual competence of a learner from 1 (no competence at all) to 10 (maximum competence). The order of the competence statements was randomized for each participant. Table 3 shows the percentage of participants who mapped a statement to a particular number.

### 3.2. Results

Table 3 shows the percentage of participants who mapped a statement to a particular number. Some statements (e.g., “limited,” “slight”) showed little agreement between participants, whilst others showed better agreement. We decided to use three statements (shown in bold) for the main studies, which are “*very low,” “moderate,”* and “*outstanding.”* These statements were selected to ensure a spread of learners' competence, good agreement between participants, and based on the average ratings and the median. However, we decided to exclude “no competence” as it was used in the explanation of the scale that participants saw in the validation experiment, and we excluded “Medium competence” and “Extreme competence” as they could be affected by a comparison with other learners in the class. More statements could be used in future studies; for example “Little competence” (or “Low competence”) and “High competence” could be used if one needed five competence statements. For our future studies, we needed only three.

## 4. Adapting the Selection of Learning Activity Knowledge Complexity to Emotional Stability and Competence

In this study, we investigate the impact of learners' emotional stability and competence on the selection of the next LA. In particular, we investigate the impact on the selection of both the knowledge taught by the LA and the prior knowledge it uses. We use three levels of competence: “very low,” “moderate,” and “outstanding.” Through an empirical study, we investigate how humans select the next LA for a learner with various levels of emotional stability and competence. Participants considered a fictional learner with certain levels of competence and emotional stability, recent and prior LA engaged in, and selected the next LA in terms of the knowledge it used and the knowledge it taught.

### 4.1. Variables

**Figure 1:**
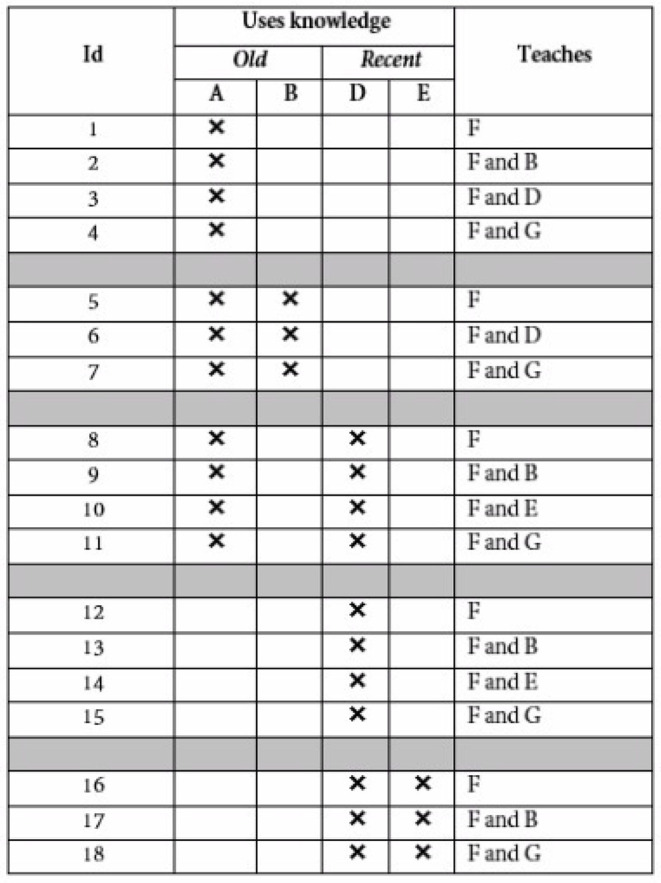
LAs as shown to participants.

The independent variables used for the study are: *Personality Trait Story:* Participants were shown a story about a learner which portrayed a personality trait. Two stories were used depicting Emotional Stability (ES) at either a low or high level. The ES stories were developed and validated by Dennis et al. (2012), Smith et al. (2019). *Learner competence:* Three levels of competence were used: very low, moderate, and outstanding. The dependent variable for the studies is *Learning activity selected:* Participants were shown a table with each row containing a LA (numbered from 1 to 18). For each LA, the table showed the PRE knowledge the LA uses, with a distinction made between old knowledge (topics A and B) and recent knowledge (D,E). It also showed the POST knowledge the LA teaches; this could contain new (F and G), old (A,B), or recent knowledge (D,E). For example, for LA 9, it indicated that it uses old knowledge A and recent knowledge D, and teaches F and B. The LAs available for selection are showed in Figure 1. Participants selected one LA, and in doing so made a choice for PRE and POST knowledge. We will use PRE and POST as the dependent variables.

### 4.2. Procedure

Participants had to pass an English fluency test (Cloze test, Taylor, 1953), to ensure that they could understand the study. Then, they were shown a description of the LA table with examples and two verification questions to ensure they had understood what the table indicated. Next, they were shown six scenarios with different learner competence *(very low, moderate, and outstanding)*, three scenarios depicting *Josh* who was high on ES and another three depicting *James* who was low on ES. They were told that the learner has previously learned topics A and B, and recently finished a LA which taught topics D and E. For each scenario, they selected the next LA for that learner from the table described before *e.g., “Which learning activity would you give to Josh to do next, if you know his competence in both old and recent knowledge is ‘Very low’?”* Next, they rated how much they think the selected LA is suited to that learner on a scale from 1 (Not at all) to 5 (Totally suited), and to what extent the selected LA would be enjoyable, would increase skills and confidence (on a scale from 1 strongly disagree to 5 strongly agree).

### 4.3. Participants

Fifty-three participants responded to the on-line survey. 24 responses were excluded from the study either because they did not pass the English test, or answered the verification questions incorrectly. Twenty-nine participants successfully completed the study (16 female, 13 male; 2 aged 18–25, 11 aged 26–40, 10 aged 36–45, 6 over 46; 8 were students, 19 were teachers, 2 were trainee-teachers).

### 4.4. Hypotheses

We hypothesized that:

- **H1**. Participants will select different LAs for high ES than low ES learners:
  – **H1.1**. They will select LAs with less complicated POST for low ES than high ES.
  – **H1.2**. They will select LAs with less complicated PRE for low ES than high ES.
- **H2**. Participants will select different LAs for learners with different competence levels:
  – **H2.1**. They will select LAs with less complicated POST for lower levels of competence.
  – **H2.2**. They will select LAs with less complicated PRE for lower levels of confidence.
- **H3**. There will be an interaction between ES and competence on LA selection.
- **H4**. Participants will rate the suitability of a selected LA and the extent to which it increases confidence differently depending on the PRE and POST. In particularly, we expect that for low ES:
  – **H4.1**. They will rate the suitability of a selected LA higher when it has less complicated POST.
  – **H4.2**. They will rate the suitability of a selected LA higher when it has less complicated PRE.
  – **H4.3**. They will rate the extent to which the selected LA increases confidence higher when it has less complicated POST.
  – **H4.4**. They will rate the extent to which the selected LA increases confidence higher when it has less complicated PRE.

### 4.5. Results

#### 4.5.1. Initial Observations on LA Selection

**Figure 2:**
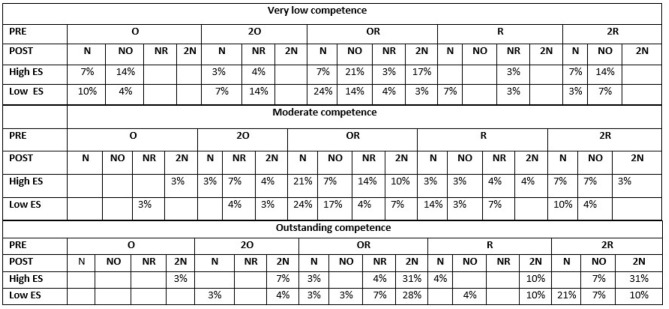
Participants' LA selection.

Figure 2 shows the proportion of participants who selected a particular LA. The LAs available for selection are summarized in the second and third row of the figure, where PRE indicates the topics the LA uses, and POST indicates the topics the LA teaches. To make the results easier to read, we use more meaningful codes here instead of the A-G participants saw. For PRE we use: (O) only one old topic, (2O) two old topics, (OR) one old and one recent topic, (R) only one recent topic, and (2R) two recent topics. For POST we use: (N) one new topic, (NO) one new topic and one old topic, (NR) one new and one recent topic and (2N) two new topics. For example, the figure shows that 7% selected a LA with PRE knowledge O and POST knowledge N for the very low competence and high ES learner. From the figure, we observe the following:

- Very low competence: Participants tended to select LAs that required old knowledge (O, 2O, OR) for both ES levels. However, participants tended to select LAs that involved learning a combination of new and old knowledge (NO) for high ES, and more just new knowledge (N) for low ES.
- Moderate competence: Participants tended to select LAs that required old knowledge for both levels of ES, but mainly OR, with O and 2O not selected much. Interestingly, a higher proportion of participants selected LAs teaching NR or 2N for high ES, whilst more selected N for low ES.
- Outstanding competence: Participants selected LAs that involved less knowledge to learn (N vs. 2N) for low ES compared to high ES.

So, overall, there is evidence of participants changing their LA selection based on ES, and were indeed selecting LAs with less complicated POST for the low ES learner. This supports hypothesis H1.1. We do not find support here for H1.2.

There is also evidence in support of hypotheses H2.1 and H2.2, as Figure 2 clearly shows that the proportion of participants selecting more complicated PRE (particularly 2R) and more complicated POST (particularly 2N) increased with an increase in competence.

#### 4.5.2. Impact of ES and Competence on PRE, POST

**Figure 3:**
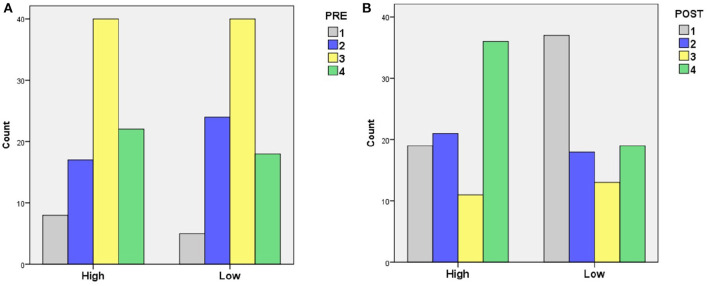
The impact of ES on **(A)** PRE and **(B)** POST knowledge.

**Figure 4:**
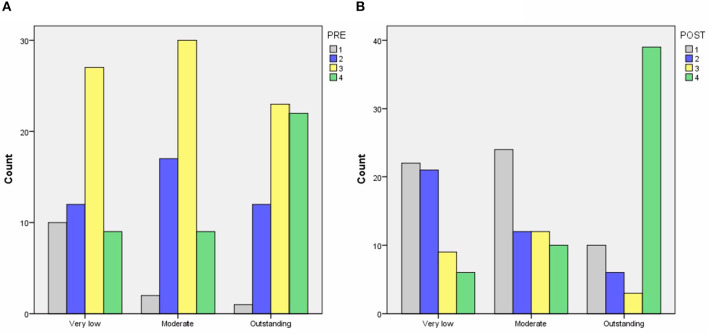
The impact of competence on **(A)** PRE and **(B)** POST knowledge.

**Figure 5:**
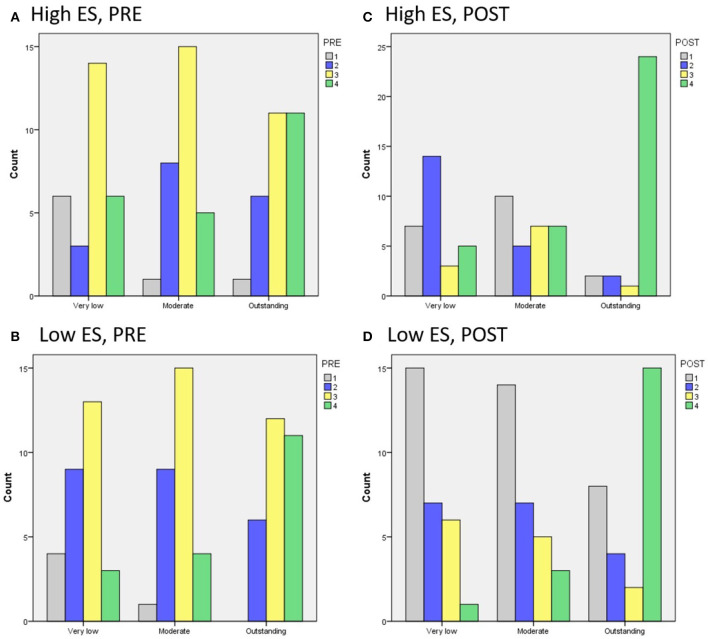
The impact of competence on PRE for **(A)** high and **(B)** low ES, and on POST for **(C)** high and **(D)** low ES.

For the statistical analysis, we coded PRE and POST in such a way that a higher number indicates more (complicated) knowledge. For PRE, we coded O=1, 2O=2, R=2, OR=3 and 2R=4, so assigning higher numbers the more (complicated) knowledge is used^2^. For POST, we coded N=1, NO=2, NR=3, 2N=4, so assigning higher numbers the more (complicated) knowledge was taught. Figures 3–5 show the overall impact of ES and competence on PRE and POST.

1. Emotional Stability: There was a significant main effect of ES on POST [*F*_(1, 168)_ = 12.3, *p* < 0.005]^3^, but not on PRE. LAs selected for high ES taught more new knowledge than LAs selected for low ES. Figure 3 shows a trend for LAs with more PRE being selected for high ES than low ES. However, the difference was small and not significant. This support hypothesis H1.2 but not H2.2.
2. Competence: There was a significant main effect of competence on both PRE and POST [*F*_(2, 168)_ = 6.0, *p* < 0.005; *F*_(2, 168)_ = 22.7, *p* < 0.0005, respectively]^4^. For POST, pairwise comparisons showed a significant difference between “very low” and “moderate” competence on the one hand, and “outstanding” competence on the other (*p* < 0.0005), with LAs with more POST selected for “outstanding” competence (mean difference = 1.24 and 1.09, respectively). For PRE, there was a significant difference only between “very low” and “outstanding” competence (*p* < 0.005), with more PRE selected for “outstanding” competence (mean difference = 0.53) (see Figure 4). This supports hypotheses H2.1 and H2.2.
3. Interaction between ES and competence: Figure 5 shows the PRE and POST per competency level for high and low ES. There was no significant interaction effect between ES and competence, so there is no evidence in support of H3.

#### 4.5.3. Suitability, Enjoyment, Increasing Skills, and Confidence

**Figure 6:**
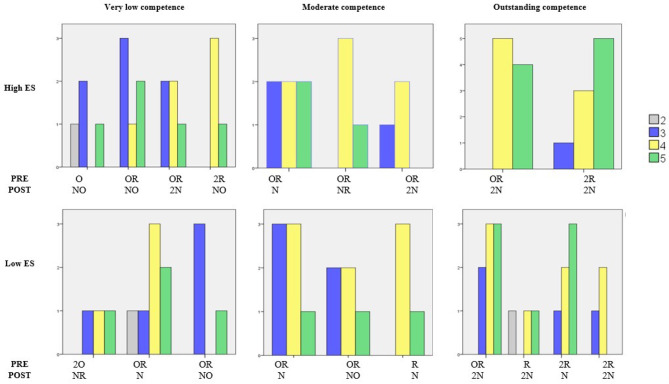
Suitability ratings of the most selected LAs.

Figure 6 shows participants' suitability ratings for the most selected LAs. Table 4 shows participants' enjoyment, skills and confidence ratings for the most selected LAs for the different levels of competence and ES. Overall, there were no significant effects of ES and competence on suitability. There was a significant effect of ES on enjoyment [*F*_(1, 168)_ = 9.6, *p* < 0.005] with a higher enjoyment rating for high ES (mean of 3.7 compared to 3.3), but not on skills and confidence. There was also a significant effect of competence on enjoyment [*F*_(2, 168)_ = 4.3, *p* < 0.05], with a higher enjoyment rating for higher competence (mean of 3.8 for outstanding competence compared to 3.5 for moderate and 3.3 for very low), but not on skills and confidence. There were no significant interaction effects. Given participants' selection of LAs (and hence the LAs for which suitability, enjoyment, skills and confidence were rated) differed based on competence and ES, we also explored this in more detail, though the number of participants is too low for statistical tests.

**Table 4:** Mean (stdev) appreciation.

**Competence**	**ES**	**Percentage (%)**	**PRE**	**POST**	**Enjoyable**	**Skills**	**Confidence**
Very low	High	21	OR	NO	3.67 (0.81)	3.83 (0.40)	3.83 (0.75)
		17	OR	2N	3.40 (1.51)	3.60 (0.89)	3.60 (0.89)
		14	O	NO	3.00 (81)	3.75 (0.50)	3.50 (0.57)
		14	2R	NO	3.50 (1.00)	4.00 (0.00)	3.25 (0.95)
	Low	24	OR	N	3.14 (1.34)	4.29 (0.48)	3.71 (1.11)
		14	2O	NR	2.25 (1.25)	3.50 (0.57)	3.75 (0.50)
		14	OR	NO	3.00 (0.81)	3.50 (0.57)	3.00 (0.81)
Moderate	High	21	OR	N	3.50 (0.83)	4.00 (0.63)	3.17 (0.75)
		14	OR	NR	4.25 (0.50)	4.25 (0.50)	4.00 (0.81)
		10	OR	2N	3.33 (0.57)	4.00 (0.00)	3.67 (0.57)
	Low	24	OR	N	3.14 (0.69)	4.14 (0.69)	3.00 (0.81)
		14	OR	NO	3.20 (1.09)	3.60 (0.89)	3.60 (0.89)
		14	R	N	4.25 (0.95)	4.50 (0.57)	5.00 (0.00)
Outstanding	High	31	OR	2N	4.11 (0.60)	4.78 (0.44)	4.33 (0.70)
		31	2R	2N	4.22 (0.83)	4.44 (0.72)	4.00 (0.86)
	Low	28	OR	2N	3.14 (0.69)	4.14 (0.69)	3.00 (0.81)
		21	2R	N	3.20 (1.09)	3.60 (0.89)	3.60 (0.89)

##### 4.5.3.1. Very low competence

For high ES, the LA which uses more recent knowledge (2R) was rated more suitable than those that used more old knowledge (O and OR) with the same POST (NO). The skills rating for this LA was also higher, whilst it confidence rating was lower. Participants may have felt that the high ES learner did not require a LA that would increase their confidence but rather their skills. For low ES, the LA that teaches less knowledge (N instead of NO) with the same PRE (OR) was rated more suitable. This LA also had a higher rating for confidence, which may mean that participants felt the low ES learner needed to gain more confidence.

##### 4.5.3.2. Moderate competence

For low ES, the LA which uses less knowledge (R) was rated more suitable than the one that uses more knowledge (OR) with the same POST (N). This LA also had a much higher rating for confidence. For high ES, the LA that teaches NR was rated more suitable than the ones teaching N or 2N, with the same PRE (OR). This LA also rated higher on the other aspects.

##### 4.5.3.3. Outstanding competence

For low ES, the LA which teaches less knowledge (N) was rated more suitable than the one that teaches more knowledge (2N) with the same PRE (2R). This LA also had a higher rating for confidence, whilst it had a lower rating for skills.

Overall, this seems to suggest that LAs that are teaching less knowledge or using less knowledge are seen as more suitable for low ES, because they may increase confidence. This provides some support for hypotheses H4.1–H4.4.

#### 4.5.4. Initial Algorithms for Adapting Learning Activity Selection Based on the Data

The main concern of this paper was to investigate how to select the next LA for a learner with a particular level of ES and competence. Using the data presented in Figure 2, three initial approaches were used to produce algorithms for selecting LAs:

1. *Most frequently chosen LA*. For each combination of competence and ES, we considered which LA was most frequently selected (see summary in Table 5). In case of outstanding competence and high ability, two LAs were chosen as often. In this case we selected the one with the same PRE as had been selected for low ES, given there had not been a significant effect of ES on PRE. This resulted in Algorithm 1.
2. *LA produced by combining the most frequently chosen PRE and the most frequently chosen POST*. For each combination of competence and ES, we considered which PRE and which POST were most frequently selected (see summary in Table 5). Using the LA which combines the most frequently selected PRE and the most frequently selected POST produced the same results as using the most frequently selected LA^5^. Hence, Algorithm 1 is already in line with the outcome of this approach and no new algorithm was produced.
3. *Top 3 LA exhibiting the largest increase in selection compared to the opposite ES case*. The differences in frequency between the most selected LAs and the second (or even third) most selected LAs tended to be relatively small. Therefore, we also considered for each combination of competence and ES, which top 3 LA showed the largest increase in frequency of selection compared to the opposite ES case. For example, for outstanding competence and high ES, 2R→2N is the top 3 LA which the largest increase in frequency (31% for high ES and only 10% for low ES). This resulted in Algorithm 2.

**Table 5:** Most frequently selected LA, PRE, and POST, and the percentage of participants who selected them, and LA with largest increase in selection.

**Competence**	**ES**	**LA (%)**	**PRE (%)**	**POST (%)**	**Largest increase LA**
Very low	High	OR → NO (21%)	OR (48%)	NO (49%)	OR → 2N
	Low	OR → N (24%)	OR (45%)	N (37%)	OR → N
Moderate	High	OR → N (21%)	OR (52%)	N (34%)	OR → NR
	Low	OR → N (24%)	OR (52%)	N (48%)	R → N
Outstanding	High	OR → 2N (31%),	OR (38%),	2N (75%)	2R → 2N
		2R → 2N (31%)	2R (38%)		
	Low	OR → 2N (28%)	OR (41%)	2N (52%)	2R → N

**Algorithm 1:**
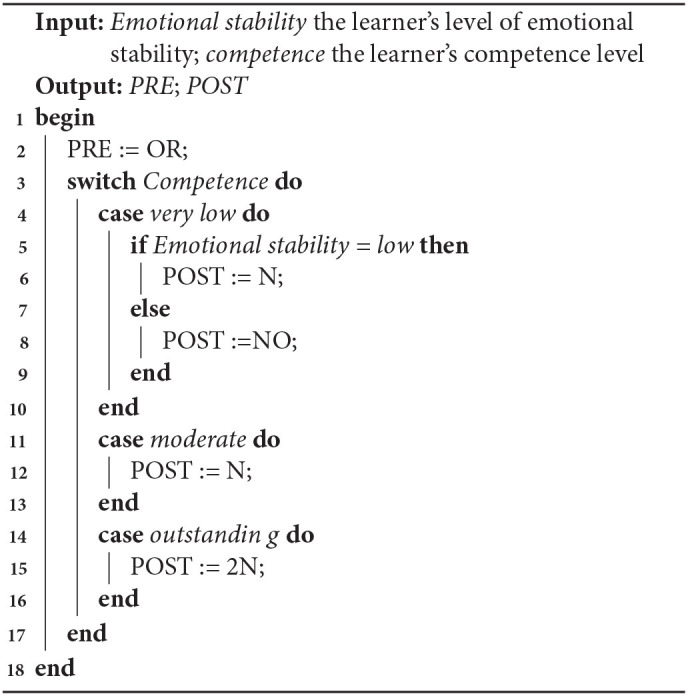
LA selection based on the most frequent LA selected

**Algorithm 2:**
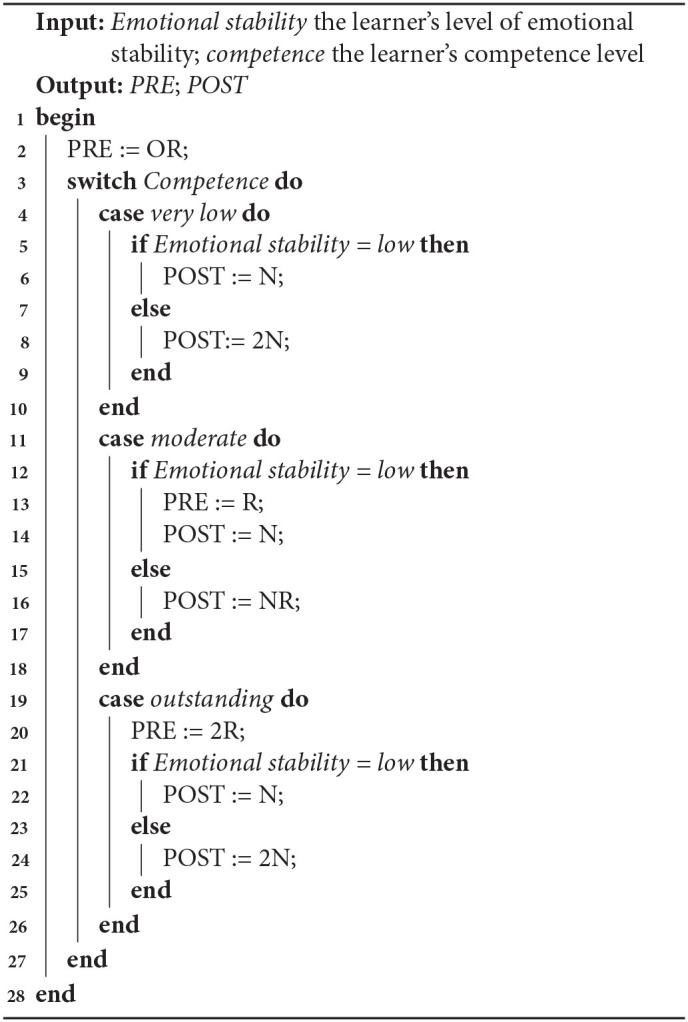
LA selection based on the largest increase in frequency

In the next section, a more complicated statistical approach will be used resulting a third algorithm, and the three algorithms will be evaluated below.

#### 4.5.5. Regression Analysis and Resulting Algorithm

Using the data of the study, two cumulative odds ordinal logistic regressions with proportional odds were run to predict the PRE and POST based on ES and competence^6^. The final model for both PRE and POST statistically significantly predicted the PRE and POST level over and above the intercept-only models [χ^2^_(2)_ = 10.458, *p* < 0.01; χ^2^_(2)_ = 41.759, *p* < 0.0005, respectively]. The odds ratio of selecting a higher POST level for learners with high ES vs. low ES is 2.707 (95% CI, 1.531–4.787), a statistically significant effect, Wald χ^2^_(1)_ = 11.740, *p* < 0.005. This supports hypothesis H1.1. An increase in competence was associated with selecting a higher POST level with an odds ratio of 2.768 (95% CI, 1.919–3.983), Wald χ^2^_(1)_ = 29.734, *p* < 0.0005. This supports H2.1 and also provides evidence that competence has slightly more impact on POST than ES. An increase in competence was also associated with selecting a higher PRE level with an odds ratio of 1.756 (95% CI, 1.240–2.487), Wald χ^2^_(1)_ = 10.059, *p* < 0.005. This supports H2.2. The odds-ratio for high ES vs. low ES for PRE was not significant, so there is again no evidence of H1.2. The model is using an interaction between ES and competence, so provides some support for H3.

The model provides coefficients to calculate a value, as well as thresholds to compare the calculated value against to produce cumulative odds for PRE and POST levels.

The model's coefficients result in the following formulae to calculate Value for PRE and POST:

- PRE:
  – 0.563 X Competence + 0.175 if ES = High
  – 0.563 X Competence if ES = Low
- POST:
  – 1.018 X Competence + 0.996 if ES = High
  – (1.018 X Competence) if ES = Low

The thresholds lead to the following formulae to calculate the natural logarithm of the cumulative odds for PRE and POST:

- PRE:
  – ln(Odds(PRE ≤ 1)) = −1.378 –Value
  – ln(Odds(PRE ≤ 2)) = 0.381 –Value
  – ln(Odds(PRE ≤ 3)) = 2.471 –Value
- POST:
  – ln(Odds(POST ≤ 1)) = 1.572 –Value
  – ln(Odds(POST ≤ 2)) = 2.643 –Value
  – ln(Odds(POST ≤ 3)) = 3.386 –Value

Using these formulae, for each combination of competence and ES we calculated:

- Value, see Table 6
- Odds(PRE ≤ d), for all PRE levels d
- Probability P(PRE ≤ d) for all PRE levels d
- P(PRE=d) for all PRE levels d, using that P(PRE ≤ 1) = P
- (PRE = 1) and P(PRE = d+1) = P(PRE ≤ d+1) –P(PRE ≤ d)
- Median PRE m such that P(PRE ≤ m) ≥ 0.5 ∧ P (PRE ≥ m) ≥ 0.5.

**Table 6:** Model predictions for PRE and POST.

**Competence**	**ES**	**PRE**	**Calculated value**	**Median PRE**	**POST**	**Calculated value**	**Median POST**
Very low	High	1 (O)	0.107	3	1 (N)	0.391	2
		2 (2O or R)	0.304		2 (NO)	0.261	
		3 (OR)	0.438		3 (NR)	0.145	
		4 (2R)	0.150		4 (2N)	0.202	
	Low	1 (O)	0.125	3	1 (N)	0.635	2
		2 (2O or R)	0.329		2 (NO)	0.200	
		3 (OR)	0.416		3 (NR)	0.079	
		4 (2R)	0.129		4 (2N)	0.086	
Moderate	High	1 (O)	0.064	3	1 (N)	0.188	3
		2 (2O or R)	0.220		2 (NO)	0.215	
		3 (OR)	0.478		3 (NR)	0.184	
		4 (2R)	0.236		4 (2N)	0.412	
	Low	1 (O)	0.075	3	1 (N)	0.386	2
		2 (2O or R)	0.246		2 (NO)	0.261	
		3 (OR)	0.471		3 (NR)	0.147	
		4 (2R)	0.206		4 (2N)	0.206	
Outstanding	High	1 (O)	0.037	3	1 (N)	0.077	3
		2 (2O or R)	0.147		2 (NO)	0.119	
		3 (OR)	0.462		3 (NR)	0.143	
		4 (2R)	0.352		4 (2N)	0.660	
	Low	1 (O)	0.044	3	1 (N)	0.185	3
		2 (2O or R)	0.168		2 (NO)	0.214	
		3 (OR)	0.473		3 (NR)	0.184	
		4 (2R)	0.313		4 (2N)	0.418	

Similar calculations were performed for POST. Table 6 shows the calculated values for all our combinations of competence and ES for PRE and POST, respectively, and how these values map onto the median PRE and POST levels. The predicted median PRE and POST levels were used to produce Algorithm 3.

**Algorithm 3:**
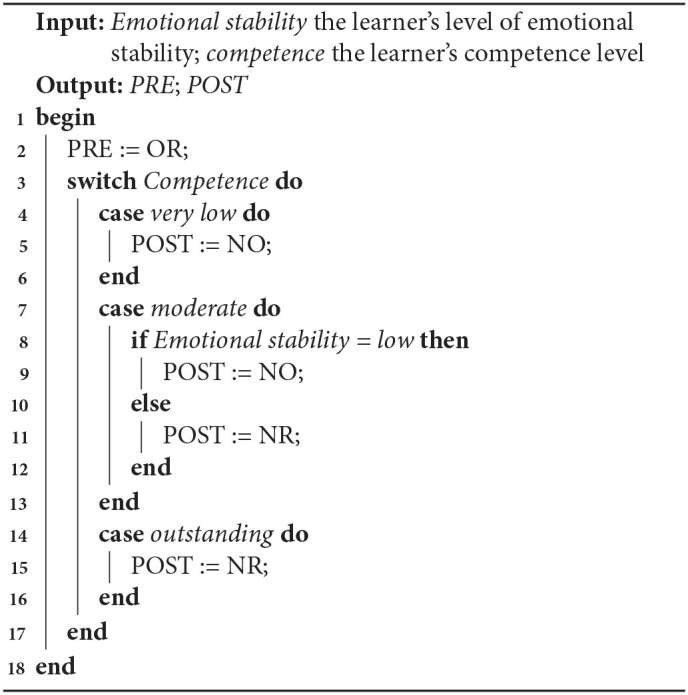
LA selection based on the regression analyses

This study investigated the impact of learner personality (emotional stability) and competence on the selection of a LA based on the knowledge it uses and the knowledge it teaches. ES and competence both impacted the selection of LAs. There were significant effects of ES on POST knowledge, and competence on both PRE and POST knowledge. A further exploratory analysis suggests that selecting LAs with less POST or PRE knowledge is better for low ES learners in terms of suitability and to increase confidence. Based on the data analysis, three algorithms have been constructed to adapt LA selection to different levels of ES and competence (see summary in Table 7).

**Table 7:** Predictions of LA selections.

**Competence**	**ES**	**LAs selection**					
		**Algorithm 1**		**Algorithm 2**		**Algorithm 3**	
		**PRE**	**POST**	**PRE**	**POST**	**PRE**	**POST**
Very low	High	OR	NO	OR	2N	OR	NO
	Low	OR	N	OR	N	OR	NO
Moderate	High	OR	N	OR	NR	OR	NR
	Low	OR	N	R	N	OR	NO
Outstanding	High	OR	2N	2R	2N	OR	NR
	Low	OR	2N	2R	N	OR	NR

## 5. Evaluation and Refinement of Algorithms

Above, we created three algorithms to adapt the selection of LAs to learner personality (ES) and competence. This section describes an evaluation of key aspects of these algorithms with teachers, resulting in a final algorithm.

### 5.1. Participants

Twenty-seven participants took part. Six were excluded from the study due to their incorrect answer to the verification question. The final sample consisted of 21 participants (11 female, 9 male, 1 non-disclosed; 9 26–35, 7 36–45, 3 over 46, and 2 prefer not to say; 10 teachers, 11 trainee-teachers).

### 5.2. Materials

We used the following materials:

1. Two stories depicting ES at either a low or high level developed by Dennis et al. (2012).
2. Three validated levels of competence: very low, moderate, and outstanding.
3. Seven LAs selected based on the three algorithms produced above (LAs 8–12, 16, 18 from Figure 1). LAs were shown as before.

### 5.3. Procedure

Ethical approval was obtained from the University of Aberdeen's Engineering and Physical Sciences ethics board. Before taking part, participants provided informed consent. Participants first provided demographic information (age, gender and occupation). They were shown two scenarios, one depicting Josh who was high on ES and another depicting James who was low on ES. They were told that the learners had previously learned topics A and B, and recently finished a learning activity which taught topics D and E. For each scenario, three questions were asked, each highlighting a different competence level (*very low, moderate, outstanding*). Participants ranked a subset of the seven LAs, based on their suitability for that learner. Table 8 shows for each level of competence and ES which LAs participants ranked, using the PRE and POST to describe the LAs. These LAs were chosen such that they included the LAs recommended by each of the three algorithms for that combination of competence and ES (as denoted in Table 8), as well as any LAs recommended by the algorithms for that level of competence but for the opposite ES.

**Table 8:** Median and average for LAs' rankings.

**Competence**	**Emotional stability**	**LAs**		**Proposed by Algorithm**	**Median**	**Average**	**Chosen for Algorithm 4**	
		**PRE**	**POST**				**PRE**	**POST**
Very low	High	OR	N		**2**	1.76	OR	NO
		OR	NO	1, 3	**2**	**1.57**		
		OR	2N	2	3	2.67		
	Low	OR	N	1, 2	**2**	1.62	OR	NO
		OR	NO	3	**2**	**1.57**		
		OR	2N		3	2.81		
Moderate	High	OR	N	1	**2**	2.29	OR	NO
		OR	NO		**2**	**2.19**		
		OR	NR	2, 3	3	2.67		
		R	N		4	2.86		
	Low	OR	N	1	**2**	2.38	OR	NO
		OR	NO	3	**2**	**2.14**		
		OR	NR		3	2.81		
		R	N	2	3	2.67		
Outstanding	High	OR	NR	3	4	3.29	2R	2N
		OR	2N	1	3	2.52		
		2R	N		**2**	2.43		
		2R	2N	2	**2**	**1.76**		
	Low	OR	NR	3	**2**	2.48	2R	N
		OR	2N	1	3	2.95		
		2R	N	2	**2**	**2.05**		
		2R	2N		3	2.52		

*Bold in Median means best median, and bold in Average means the best average among the LAs rankings*.

### 5.4. Research Questions

We investigated the following research questions:

1. For each level of learner competence and ES, how highly are the selected LAs by Algorithm 1, Algorithm 2, and Algorithm 3 ranked by the teachers, and which LA is ranked highest?
2. Which algorithm matches the rankings of the teachers best?
3. What modifications are needed to the best algorithm to be in line with teachers' preferences?

### 5.5. Results

**Figure 7:**
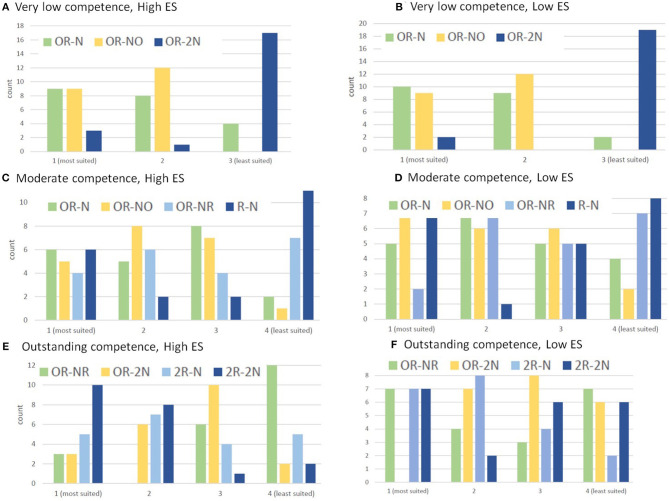
Teachers' rankings for LAs for **(A,B)** very low competence, **(C,D)** moderate competence, and **(E,F)** outstanding competence, for **(A,C,E)** high ES and **(B,D,F)** low ES.

Table 8 and Figure 7 show the results of the ranking. We calculated both the average rank and the median rank.

#### 5.5.1. Very Low Competence

For high ES, the teachers' ranking is best for OR→NO, in line with the predictions of Algorithms 1 and 3. For low ES, the teachers ranking is also best for OR→NO, matching the prediction of Algorithm 3. The prediction by Algorithm 2 did badly in the high ES case, with teachers clearly preferring less complicated LAs than Algorithm 2 had predicted. In fact LAs that involved more new knowledge to learn (2N) were deemed to be the least suited LAs for both ES levels. OR→NO and OR→N did about equally well in the low ES case, so overall the predictions by Algorithm 1 are also good. The teachers clearly where in two minds on whether adaptation to ES would be a good idea for learners with very low competence. Follow on studies measuring learners' attainment and motivation should show whether it is better to use OR→NO or OR→N for low ES learners.

#### 5.5.2. Moderate Competence

For high ES, the teachers' ranking is best for OR→NO. This is not predicted by any of the algorithms. Algorithm 1 predicted a less complicated LA, namely OR→N, whilst Algorithms 2 and 3 predicted a more complicated LA, namely OR→NR. Teachers went for an LA in between, with the ranking of that LA close to that predicted by Algorithm 1. For low ES, the teachers' ranking is best for OR→NO, in line with the prediction of Algorithm 3. Algorithm 2 did badly for both levels of ES. For moderate competence, there is no evidence of adapting to ES levels.

#### 5.5.3. Outstanding Competence

For high ES, the teachers' ranking is best for 2R→2N, in line with the prediction by Algorithm 2. We recall that two LAs scored equally well when constructing Algorithm 1. We selected OR→2N at the time, given the lack of a statistically significant effect of PRE. The alternative was 2R→2N. The teachers clearly preferred the latter one. For low ES, the teachers' ranking is best for 2R→N, again in line with the prediction of Algorithm 2, and showing that teachers are adapting their rankings based on ES, using less new knowledge to learn for the low ES learner. Overall, for outstanding competence, Algorithm 2's predictions were perfect. For both levels of ES, teachers ranked the two LAs that required only recent knowledge higher than the two LAs that required a combination of old an recent knowledge, showing an inclination to only use recent knowledge for outstanding competence.

### 5.6. Refining the Algorithms

We did not find that one algorithm performed better than the others. Algorithm 3 performed best for the Very low competence case (and Algorithm 1 almost equally well), and also for low ES in the Moderate competence case. In contrast, Algorithm 2 performed best for the Outstanding competence case, but badly in the other ones. We decided to produce a new algorithm, combing elements from Algorithms 3 and 2. Table 8 shows the selections of LAs made for Algorithm 4, which were based on the best median rankings by the teachers. The resulting algorithm is shown in Algorithm 4.

**Algorithm 4:**
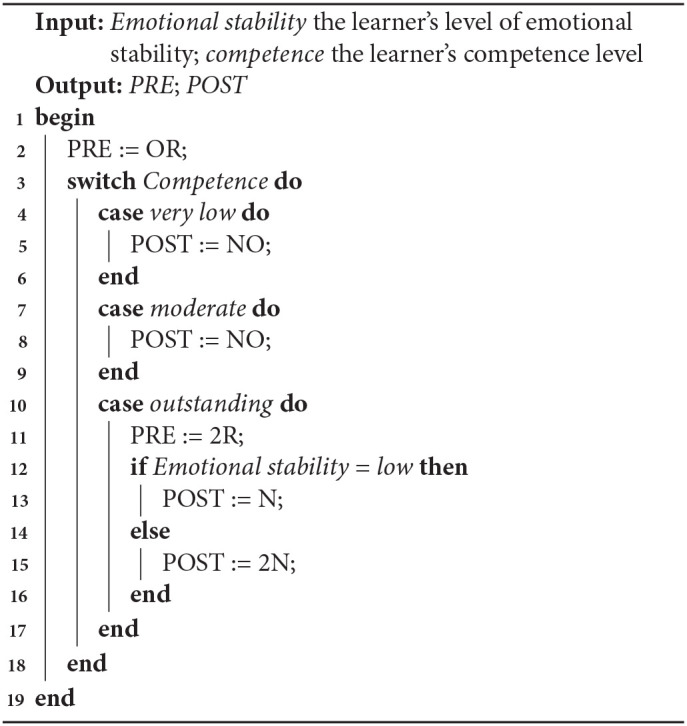
LA selection based on teachers' preferences

## 6. Conclusion

This paper investigated the impact of learner personality (emotional stability) and competence on the selection of a LA based on the knowledge it uses and the knowledge it teaches. We also investigated the extent to which the selected LAs are perceived to be enjoyable and to increase learners' confidence and skills. ES and competence both impacted the selection of LAs. There were significant effects of ES on POST knowledge, and competence on both PRE and POST knowledge. A further exploratory analysis suggests that selecting LAs with less POST or PRE knowledge is better for low ES learners in terms of suitability and to increase confidence.

Based on the data analysis, an algorithm has been constructed to adapt LA selection to different levels of ES and competence. we obtained four algorithms for adapting LA selection to learners' personality and competence. Algorithms 3 and 4 are the most promising to investigate further, with Algorithm 4 best matching the teachers' preferences, and Algorithm 3 being most aligned to the teachers' preferences from the algorithms based on the data in study 4. These algorithms can be used in an Intelligent Tutoring System, or, as we recommend in future work, can be used as a basis for further research. In addition, we obtained an insight into how teachers adapt LA selection and how this matches the algorithms developed. We found evidence that teachers take emotional stability into account when selecting different LAs.

This paper has several limitations and opportunities for future work. First, we did not measure *actual* enjoyment, increase in confidence and increase in skills, but perceptions of those. Studies with learners and real learning tasks are needed to investigate actual impact. Second, the studies in this paper used an abstract notation for learning topics, using letters, such as A, E to indicate which concepts are needed to be known to study something, and which concepts are learned in an activity. This was done on purpose, so that we could study learning activity selection without participants' preconceived ideas about difficulty level of individual concepts and learning domains interfering. However, clearly further studies need to show to what extent what was learned in this paper can be generalized to real learning topics. Further studies are also needed to investigate the possible impacts of learning domains. Third, our algorithm requires a certain structure of the learning activities, namely what is taught (i.e., learning outcomes) and what is used (i.e., prerequisites) in a learning activity. It also requires a learner model in terms of these outcomes, so that we know what a learner has already studied. This may limit its applicability, however, the use of learning outcomes (and also prerequisites) is well-established, and strongly advocated in educational science (Kennedy, 2006). Fourth, we only investigated three levels of competence and ES only at the high and low level. The competence level validation reported in this paper would allow investigating another two levels. It would also be interesting to investigate finer gradations of ES. Fifth, other learner characteristics could be investigated, for example, the impact of learner goals and interests, or as advocated by Zhu et al. (2019) participation levels. As initial research by Adamu Sidi-Ali et al. (2019) showed that cultural background may impact desired learner emotional support, we would also like to investigate whether cultural background should matter for learning activity selection. Sixth, we did not consider other personality traits. Based on previous research (Okpo et al., 2018), we expect learner self-esteem to also matter. Seventh, this paper does not consider how long ago previous topics were studied. A forgetting model will be needed to take into account the likelihood that a learner still masters a topic or that a topic may need to be used in order to prevent forgetting (see Ilbeygi et al., 2019 for an overview and recent work on forgetting models). Eight, this paper only considered learning activity selection for individual learners. This becomes an even more complicated issue when learning activities need to be selected for groups of learners for a collaborative learning experience. Finally, we only considered PRE and POST knowledge, but did not explicitly address difficulty levels.

## Data Availability Statement

The datasets generated for this study are available on request to the corresponding author.

## Ethics Statement

The studies involving human participants were reviewed and approved by a University of Aberdeen ethics committee. The participants provided their written informed consent to participate in the studies.

## Author Contributions

MA and JM designed and analyzed the studies, and wrote the paper. NB contributed to the study design and helped to improve the paper.

### Conflict of Interest

The authors declare that the research was conducted in the absence of any commercial or financial relationships that could be construed as a potential conflict of interest.

## Footnotes

^1^This includes a debate about whether learning styles are a valid construct to consider at all Kirschner (2017). Our own research in Alhathli et al. (2017) in fact showed little impact of learning styles.

^2^As it is hard to say whether 2O or R requires more knowledge, we coded them the same.

^3^A similar significant effect was found using a non-parametric test.

^4^Similar significant effects were found using non-parametric tests.

^5^Similarly to the discussion above, for outstanding competence and high ES, the most frequently selected PRE could also have been 2R instead of OR.

^6^ES was used as a factor. Competence was used as a ordinal co-variate, with competence coded 1-3 for very low till outstanding.
